# Supplementary material for: Negative spin Hall magnetoresistance of normal metal/ferromagnet bilayers
Source: Nat Commun. 2020 Jul 17;11:3619. doi: 10.1038/s41467-020-17463-3 (PMC7367820; doi:10.1038/s41467-020-17463-3)
Supplement: Supplementary file 1 — Supplementary Information [file 41467_2020_17463_MOESM1_ESM.pdf]

Supplementary Information for "Negative spin Hall magnetoresistance of normal metal/ferromagnet bilayers"

Kang *et al.*

Supplementary Note 1. Angle-dependent magnetoresistance

We measured the angle-dependent magnetoresistance (ADMR) of the Ta/NiFe, Pt/NiFe and NiFe samples, by rotating an external magnetic field of 9 T (1.3 T) in the xz-plane (xy-plane) using a DC current of 100 μA applied in the x direction. Supplementary Figure 1 shows the ADMR as a function of α (γ) angle for Ta(3 nm)/NiFe(t_F), Pt(3 nm)/NiFe(t_F), and NiFe(t_F) samples. The result show that the signs of ADMR versus α and γ angles remain the same irrespective of t_F. It is distinct from the SMR results with respect to the angle β (in yz-plane) in the main text, in which the sign inversion is observed as t_F increases. The ADMRs vs. α, β, and γ angle are summarized in Supplementary Figure 1g – 1i, respectively.

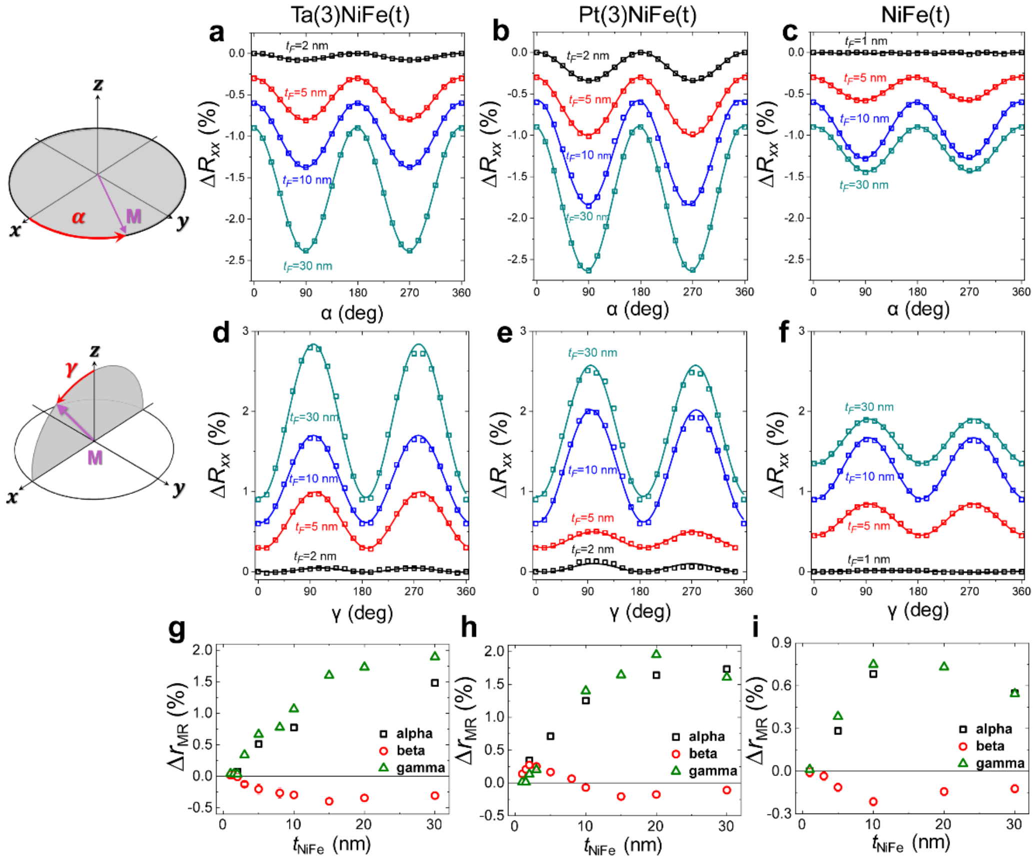

Supplementary Figure 1. Angle-dependent magnetoresistance (ADMR) of NM/NiFe (t_F) samples. a-c, ADMR vs. $\boldsymbol{\alpha}$ angle for Ta(3)/NiFe(t_F) **(a)**, Pt(3)/NiFe(t_F) **(b)**, and NiFe(t_F) **(c)**. **d-f,** ADMR vs. $\boldsymbol{\gamma}$ angle for Ta(3)/NiFe(t_F) **(d)**, Pt(3)/NiFe(t_F) **(e)**, and NiFe(t_F) **(f)**. **g-i,** ADMRs as a function of t_F for Ta(3)/NiFe(t_F) **(g)**, Pt(3)/NiFe(t_F) **(h)**, and NiFe(t_F) **(i)**. The angles $\boldsymbol{\alpha}$ and $\boldsymbol{\gamma}$ are defined in the schematics. The ADMR is defined as $\Delta \boldsymbol{R}_{xx} = [\boldsymbol{R}_{xx}(\boldsymbol{\alpha},\boldsymbol{\gamma}=\mathbf{0}) - \boldsymbol{R}_{xx}(\boldsymbol{\alpha},\boldsymbol{\gamma})]/\boldsymbol{R}_{xx}(\boldsymbol{\alpha},\boldsymbol{\gamma}=\mathbf{0})$.

Supplementary Note 2. Effect of the capping layer

For the experimental results shown in the main text, we have used a MgO/Ta capping layer. According to Supplementary Reference [1], TaO_x layer may also generate a spin current, which may transmit through a thin MgO layer into NiFe layer. To verify the effect of the top MgO/Ta layer, we investigated two additional samples; one is a substrate/NiFe(5 nm)/Ta(3 nm)/MgO(1.6 nm)/Ta structure where the stacking order of Ta and NiFe is reversed, and the other is a Ta(3 nm)/NiFe(5 nm)/MgO(10 nm)/Ta structure where the MgO layer (10 nm) is thick enough to prevent spin current transmission from the top MgO/Ta interface. Supplementary Figure 2 shows the SMR results of the two samples, compared to that of a reference sample of Ta(3 nm)/NiFe(5 nm)/MgO(1.6 nm)/Ta structure, demonstrating that SMR values are very similar within an error of 5%, irrespective of the sample structures. This result suggests that the top MgO/Ta layer does not mainly contribute to the negative SMR in Ta/NiFe bilayers.

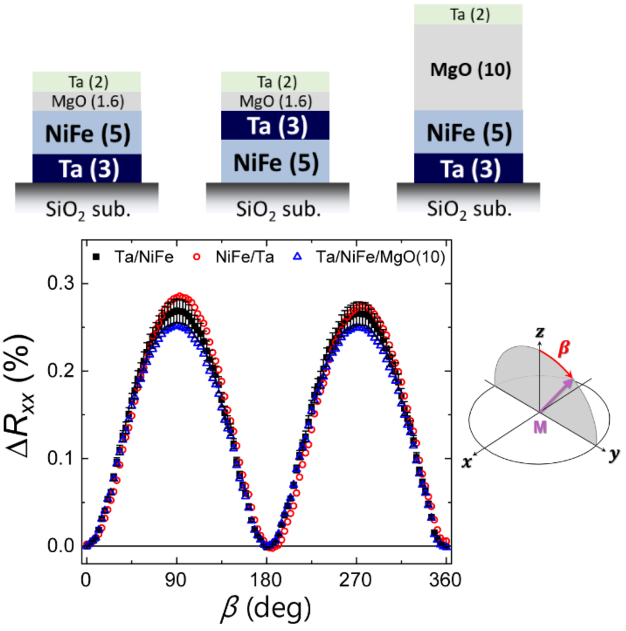

Supplementary Figure 2. SMR in various samples of Ta(3 nm)/NiFe(5 nm)/MgO(1.6 nm)/Ta, Si/SiO_2 substrate/NiFe(5 nm)/Ta(3 nm)/MgO(1.6 nm)/Ta, and Ta(3 nm)/NiFe(5 nm)/MgO(10 nm)/Ta. SMR is defined as $\Delta R_{xx} = [R_{xx}(\beta = 0) - R_{xx}(\beta)]/R_{xx}(\beta = 0)$, where β is defined in the schematic figure.

Supplementary Note 3. Thickness dependence of the longitudinal resistivities

Supplementary Figure 3a shows experimentally measured thickness dependence of $1/R_{xx}$ for Ta/NiFe and Pt/NiFe samples (w and l stand for width and length of the devices, respectively). From these results and the parallel circuit model, we estimate $\rho_{\mathrm{Pt}} \approx 40\ \mu\Omega\mathrm{cm}$, $\rho_{\mathrm{Ta}} \approx 300\ \mu\Omega\mathrm{cm}$ and longitudinal resistivity of a NiFe layer as shown in Supplementary Figure 3b.

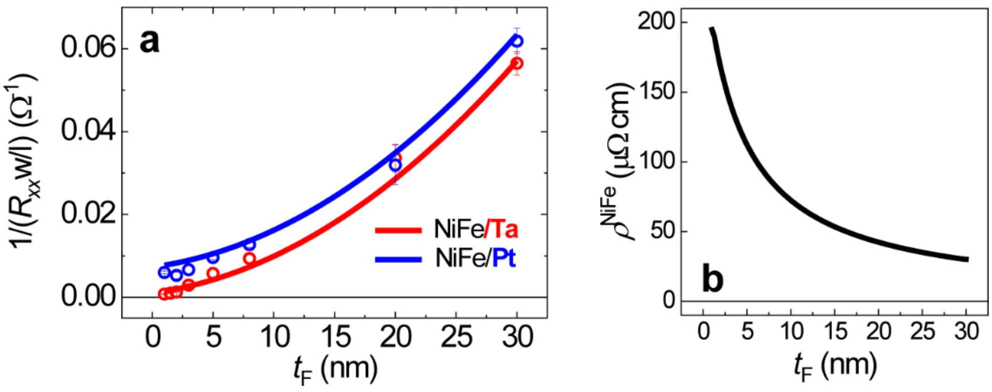

Supplementary Figure 3. Longitudinal resistivities. a, Measured data of $1/R_{xx}$ vs. t_{F} in Ta/NiFe and Pt/NiFe samples. **b,** Estimated longitudinal resistivity of a NiFe layer from the parallel circuit model. The error bars in **a** are from the statistical variation of the longitudinal resistivities.

Supplementary Note 4. Existence of an additional source for the negative Δr_{MR}

In Fig. 1f and 1h in the main manuscript, we observe that Δr_{MR} in both Ta/NiFe sample and single-layer NiFe sample are negative, and the absolute value of Δr_{MR} in Ta/NiFe sample is larger than that in single-layer NiFe sample. Based on the parallel circuit model, we show that the larger Δr_{MR} in Ta/NiFe sample implies that there exists another origin of negative Δr_{MR}. While the parallel circuit model is crude for quantitative calculation of Δr_{MR}, the argument on the existence of an additional source is valid in our experimental regime (see below).

In the parallel circuit model, the field-dependent resistances of the NM/FM bilayer sample are

$$R_{xx}(H_y) = R_0 = \frac{R_{\mathrm{F}}R_{\mathrm{N}}}{R_{\mathrm{F}} + R_{\mathrm{N}}}, \quad R_{xx}(H_z) = R_0' = \frac{R_{\mathrm{F}}'R_{\mathrm{N}}'}{R_{\mathrm{F}}' + R_{\mathrm{N}}'},$$

where $R_F' = R_F + \Delta R_F$, $R_N' = R_N + \Delta R_N$. ΔR_F and ΔR_N are the resistance changes of single FM and NM layers, respectively. If we neglect the ISOC effect, we have

$$R_{xx}(H_z) = \frac{(R_{\mathrm{F}} + \Delta R_{\mathrm{F}})(R_{\mathrm{N}} + \Delta R_{\mathrm{N}})}{R_{\mathrm{F}} + R_{\mathrm{N}} + \Delta R_{\mathrm{F}} + \Delta R_{\mathrm{N}}} \approx R_0 + R_0^2\left(\frac{\Delta R_{\mathrm{F}}}{R_{\mathrm{F}}^2} + \frac{\Delta R_{\mathrm{N}}}{R_{\mathrm{N}}^2}\right).$$

Then ΔR_{MR} of the bilayer is

$$\Delta R_{MR} = R_{xx}(H_z) - R_{xx}(H_y) = R_0^2\left(\frac{\Delta R_{\mathrm{F}}}{R_{\mathrm{F}}^2} + \frac{\Delta R_{\mathrm{N}}}{R_{\mathrm{N}}^2}\right).$$

In terms of MR ratio, we have

$$\Delta r_{MR} = \frac{R_0}{R_{\mathrm{F}}}\Delta r_{\mathrm{F}} + \frac{R_0}{R_{\mathrm{N}}}\Delta r_{\mathrm{N}},$$

where $\Delta r_{\mathrm{MR}} = \Delta R_{\mathrm{MR}}/R_0$, $\Delta r_{\mathrm{F}} = \Delta R_{\mathrm{F}}/R_{\mathrm{F}}$, and $\Delta r_{\mathrm{N}} = \Delta R_{\mathrm{N}}/R_{\mathrm{N}}$. We note that (i) Δr_{N} originates from the bulk spin Hall effect of NM and is positive, (ii) both Δr_{MR} and Δr_{F} are negative, and (iii) the longitudinal resistance of NM/FM(t_{F}) bilayer is smaller than that of the single FM layer with thickness t_{F} (R_0/R_{F} <1). From (i)-(iii), we have following inequality

$$|\Delta r_{\mathrm{MR}}| < |\Delta r_F|.$$

However, the experimental data in Fig. 1f and 1h show that $|\Delta r_{\mathrm{MR}}|$ is about twice as large as

$|\Delta r_{\mathrm{F}}|$, which violates the above inequality. Thus, we conclude that there is another source for negative Δr_{MR} in our Ta/NiFe sample.

Supplementary Note 5. SMR in Cu/NiFe bilayers

We investigate SMR in Cu/NiFe bilayers, in which the bulk spin Hall effect is negligible. We fabricated Cu(t_{Cu})/NiFe(5 nm) bilayers with various Cu thicknesses t_{Cu} from 1 nm to 12 nm. Supplementary Figure 4a shows the angle-dependent MR of Cu/NiFe bilayers, where the angle β is defined in the schematic. Supplementary Figure 4b summarizes the SMR as a function of t_{Cu}. It is found that the SMR decreases with increasing t_{Cu} and is negative for all samples.

The overall tendency of decreased SMR with increasing t_{Cu} can be explained by the reduced GSE of NiFe due to the current shunting through the Cu layer. In order to check if the current shunting effect explains the observed t_{Cu} dependence of SMR quantitatively, we fit the experimental results with considering the only GSE, estimated from the parallel circuit model (a red curve). We find some deviations between the experimental results and fitting curve, implying that the Cu/NiFe interface may generate a spin current. This possibility of interface-generated spin current at the Cu/NiFe interface may be supported by the additional experiment, demonstrating non-negligible damping-like effective field (B_{DLT}) in Cu/NiFe bilayers. The experimental data for the SOT measurement will be presented in the Supplementary Note 6.

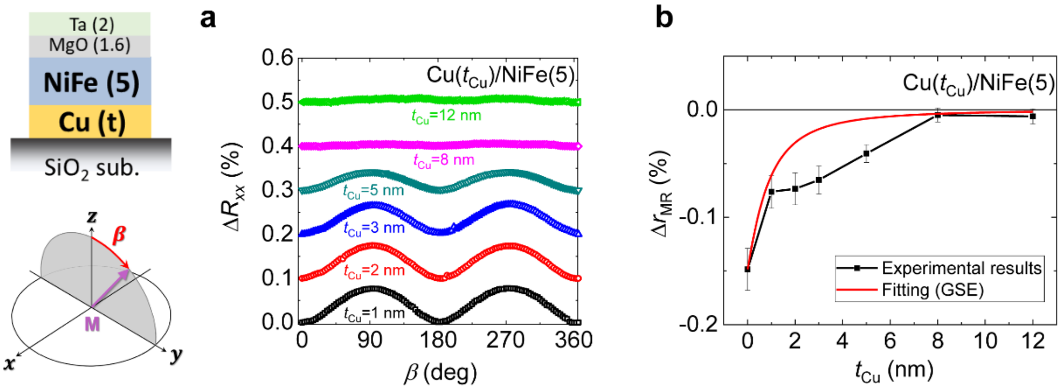

Supplementary Figure 4. Cu-thickness dependence of SMR in Cu/NiFe. a, β-angle dependence of MR **[**$\Delta R_{xx} = [R_{xx}(\beta = 0) - R_{xx}(\beta)]/R_{xx}(\beta = 0)$**]** in Cu($t_{Cu}$)/NiFe(5). β is indicated in the left schematic. **b,** Cu thickness (t_{Cu}) dependence of SMR ratio (Δr_{MR}). Solid red curve describes fitting result with only considering GSE of NiFe. The error bar in **b** are from the statistical variation of the SMR.

Supplementary Note 6. Harmonic measurements of SOT

We measured current-induced SOT in Ta(3 nm)/NiFe(5 nm), NiFe(5 nm), and Cu(3 nm)/NiFe(5 nm) samples using in-plane harmonic Hall resistance measurements [2]. As schematically depicted in top right of Supplementary Figure 5, the 1st and 2nd harmonic Hall resistances ($R_{xy}^{1\omega}, R_{xy}^{2\omega}$) are measured by rotating the sample (azimuthal angle φ) under a fixed in-plane magnetic field strength B_{ext} and an AC current I_{AC}. The $R_{xy}^{2\omega}$ is expressed as

$$R_{xy}^{2\omega}(\varphi) = \left(R_{\text{AHE}}\frac{B_{\text{DLT}}}{B_{\text{eff}}} + R_{\nabla T}^{2\omega}\right)\cos\varphi + 2R_{\text{PHE}}\frac{B_{\text{FLT}}+B_{\text{Oe}}}{B_{\text{ext}}}(2\cos^3\varphi - \cos\varphi).$$

Here, R_{AHE} and R_{PHE} are the anomalous Hall and planar Hall resistances, respectively; B_{DLT}, B_{FLT}, and B_{Oe} are the damping-like effective field, the field-like effective field, the Oersted field, respectively; B_{eff} is the effective magnetic field including the demagnetization field B_{dem} and the anisotropy field B_{ani} of FM ($B_{\text{eff}} = B_{\text{ext}}+B_{\text{dem}}-B_{\text{ani}}$); $R_{\nabla T}^{2\omega}$ is the thermoelectric contribution to $R_{xy}^{2\omega}$. Supplementary Figures 5a-l show the measurement results of $R_{xy}^{1\omega}$ and $R_{xy}^{2\omega}$ versus φ curves for Ta/NiFe, NiFe, and Cu/NiFe samples. The $R_{xy}^{1\omega}$ exhibits a typical 2φ dependence due to the planar Hall effect (Supplementary Figure 5a-c), while the $R_{xy}^{2\omega}$ has both cosφ and ($2\cos^3\varphi - \cos\varphi$) components (Supplementary Figure5d-f), which are separately plotted in Supplementary Figure5g-i and Supplementary Figure5j-l, respectively. We extract the B_{DLT} from the slope of cosφ component of $R_{xy}^{2\omega}$ versus $1/B_{\text{eff}}$ curves (Supplementary Figure5m). It is observed that Ta/NiFe bilayer exhibits a sizable slope, resulting in B_{DLT} of ~ –0.45 mT/($1.0 \times 10^7 Am^{-2}$). On the other hand, NiFe single layer shows a negligible slope and thereby vanishingly small B_{DLT}. Interestingly, Cu/NiFe sample also exhibits a noticeable slope, but its sign is opposite to that of the Ta/NiFe sample, resulting in B_{DLT} of ~ +0.32 mT/($1.0 \times 10^7 Am^{-2}$). This demonstrates that a spin current is generated even in Cu/NiFe bilayer, which we attribute to the ISOC effect.

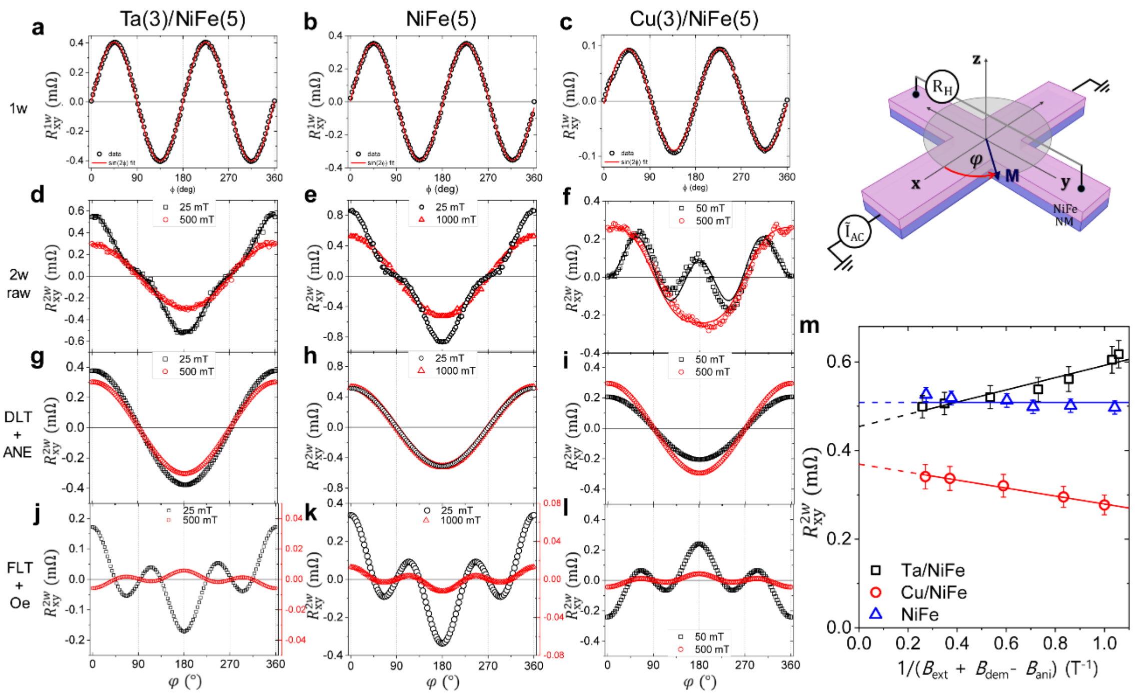

Supplementary Figure 5. Harmonic Hall SOT measurements in Ta/NiFe, NiFe and Cu/NiFe. Top right schematic indicates the detail measurement configuration. **a-c**, $\boldsymbol{R_{xy}^{1\omega}}$ versus $\boldsymbol{\varphi}$ curves for Ta/NiFe (**a**), NiFe (**b**), and Cu/NiFe (**c**). The solid line is a sin2$\boldsymbol{\varphi}$ fit to the experimental data. **d-f**, $\boldsymbol{R_{xy}^{2\omega}}$ versus $\boldsymbol{\varphi}$ curves for Ta/NiFe (**d**), NiFe (**e**), and Cu/NiFe (**f**). The solid line is a $\mathbf{cos\varphi + (2cos^3\varphi - cos\varphi)}$ fit of the experimental data. **g-i,** The cos$\boldsymbol{\varphi}$ component of $\boldsymbol{R_{xy}^{2\omega}}$ versus $\boldsymbol{\varphi}$ curves for Ta/NiFe (**g**), NiFe (**h**), and Cu/NiFe (**i**). **j-l,** $\mathbf{(2cos^3\varphi - cos\varphi)}$ components of $\boldsymbol{R_{xy}^{2\omega}}$ versus $\boldsymbol{\varphi}$ curves for Ta/NiFe (**j**), NiFe (**k**), and Cu/NiFe (**l**). **m**, The $\mathbf{cos\varphi}$ component of $\boldsymbol{R_{xy}^{2\omega}}$ versus $1/B_{eff}$ curves. The error bars in **m** are from the linear fitting to determine.

Supplementary Note 7. General symmetry analysis for interconversion between charge current and spin current

To describe the interconversion process between charge and spin, we write down the following linear response relation,

$$\begin{pmatrix} I_{\mathrm{c},x} \\ I_{\mathrm{s},z} \end{pmatrix} = L \begin{pmatrix} -\partial_x \mu/e \\ -\partial_x \mu_{\mathrm{s}}/2e \end{pmatrix}, \qquad L = \begin{pmatrix} L_{\mathrm{c}} & L_{\mathrm{cs}}^{\mathrm{T}} \\ L_{\mathrm{sc}} & L_{\mathrm{s}} \end{pmatrix}, \tag{1}$$

where L is the linear response matrix, and $L_{\mathrm{c}}(L_{\mathrm{s}})$ is linear response coefficient (matrix) for charge (spin) transport, $L_{\mathrm{cs}}(L_{\mathrm{sc}})$ amounts to the spin-to-charge (charge-to-spin) conversion by SHE, AHE, and ISOC. The induced charge current with the second-order approximation is given by

$$\Delta I_{\mathrm{c},x} \propto L_{\mathrm{cs}} \cdot L_{\mathrm{sc}}. \tag{2}$$

For the bulk SHE, we have $L_{\mathrm{sc}} = (0, \sigma_{\mathrm{SH}}, 0)$ for charge-to-spin conversion process, where σ_{SH} is the spin Hall conductivity. The Onsager reciprocity relation states that

$$L_{\mathrm{cs}} = (0, -\sigma_{\mathrm{SH}}, 0). \tag{3}$$

Because the time-reversal conjugate changes nothing but a spin degree from the charge-to-spin conversion, the sign of σ_{SH} in L_{cs} is the opposite to that in L_{sc}. For the bulk AHE, we have

$$L_{\mathrm{cs}} = \left(0, -\zeta \sigma_{\mathrm{AH}} m_y, 0\right), \qquad L_{\mathrm{sc}} = \left(0, \zeta \sigma_{\mathrm{AH}} m_y, 0\right), \tag{4}$$

where ζ describes the spin polarization of spin-polarized charge current and σ_{AH} is the anomalous Hall conductivity [3,4]. We note that the time-reversal conjugate changes the sign of ζ and m_y. The interface generated spin current flowing out-of-plane is

$$\mathbf{j}_{\mathrm{s}} = j_{\mathrm{f}} \hat{\mathbf{s}} + j_{\mathrm{p}} \hat{\mathbf{m}} \times \hat{\mathbf{s}}, \tag{5}$$

where $\hat{\mathbf{s}} = \hat{\mathbf{z}} \times \mathbf{E} = \hat{\mathbf{y}}$ [5]. Since $\hat{\mathbf{m}}$ lies on the *yz*-plane in our experiment, we have $L_{\mathrm{sc}} = \left(-j_{\mathrm{p}} m_z, j_{\mathrm{f}}, 0\right)$ for ISOC. Because the m_z and $\hat{\mathbf{s}}$ (the direction of the Rashba spin-orbit field) changes the sign under the time-reversal conjugate, we obtain

$$L_{\mathrm{cs}} = \left(j_{\mathrm{p}} m_z, j_{\mathrm{f}}, 0\right). \tag{6}$$

With above equations, we are able to express the charge current originating from various charge-to-spin conversions and their inverse effects. For instance, the additional charge current

from the SHE and ISOC are

$$(L_{\rm cs}^{\rm SHE} + L_{\rm cs}^{\rm ISOC}) \cdot (L_{\rm sc}^{\rm SHE} + L_{\rm sc}^{\rm ISOC}) = j_{\rm f}^2 - j_{\rm p}^2 m_z^2 - \sigma_{\rm SH}^2. \quad (7)$$

Supplementary Equation (7) shows three contributions each of which comes from a combined action of a process and its inverse. Therefore, there is no SHE-ISOC cross contribution. This no bulk-interface contribution is caused by the following two reasons: (i) SHE is nothing to do with the x component. Therefore, $j_{\rm p}$ does not contribute to cross terms. (ii) For $j_{\rm f}$, its time-reversal property is the opposite to that of $\sigma_{\rm SH}$, thus $\sigma_{\rm SH} j_{\rm f}$ for one process is canceled by $-\sigma_{\rm SH} j_{\rm f}$ for the reciprocal process. By the same reasons, there is no cross contribution between AHE and ISOC. However, the situation is quite different for those between SHE and AHE. Because the time-reversal properties of $\sigma_{\rm SH}$ and $\zeta \sigma_{\rm AH} m_y$ are even, the additional charge current

$$(L_{\rm cs}^{\rm SHE} + L_{\rm cs}^{\rm AHE}) \cdot (L_{\rm sc}^{\rm SHE} + L_{\rm sc}^{\rm AHE}) = -(\sigma_{\rm SH} + \zeta \sigma_{\rm AH} m_y)^2, \quad (8)$$

includes a cross term $-2\zeta \sigma_{\rm SH} \sigma_{\rm AH} m_y$.

The realistic situation is more complicated because the regions where the electrons flow for each case are different (NM, FM, interface) and there are interface conductances between them. Although the full solution is complicated, the absence of the cross term by using this simple argument based on the time-reversal property should be valid. In Supplementary Note 8, we derive the full solution including NM, FM, and NM/FM interface, based on the drift-diffusion formalism.

Supplementary Note 8. Extended spin drift-diffusion model calculation

Here we derive analytic expressions for the Δr_{MR} originating from various physical origins using the drift-diffusion formalism. The schematic illustration of the model system is given in Supplementary Figure6.

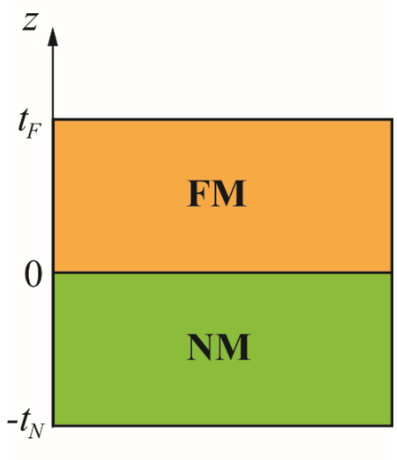

Supplementary Figure 6. Schematic illustration of the model system.

A. Drift-diffusion model calculation

In a thin FM layer, the longitudinal charge and transverse spin current densities are given by [3,4]

$$j_x^{\mathrm{F}}(\mathbf{m}=\hat{\mathbf{z}}) = \sigma_{\mathrm{F}} E_x - \zeta \theta_{\mathrm{AH}} j_y^{\mathrm{s,F}}(\mathbf{m}=\hat{\mathbf{z}}),$$

$$j_y^{\mathrm{s,F}}(\mathbf{m}=\hat{\mathbf{z}}) = \zeta \sigma_{\mathrm{AH}} E_x, \qquad j_z^{\mathrm{s,F}}(\mathbf{m}=\hat{\mathbf{z}}) = -\frac{\sigma_{\mathrm{F}}}{2e}\partial_z \mu_{\mathrm{s}}^{\mathrm{F}},$$

$$j_x^{\mathrm{F}}(\mathbf{m}=\hat{\mathbf{y}}) = j_0^{\mathrm{F}} + \zeta \theta_{\mathrm{AH}} j_z^{\mathrm{s,F}}(\mathbf{m}=\hat{\mathbf{y}}),$$

$$j_z^{\mathrm{s,F}}(\mathbf{m}=\hat{\mathbf{y}}) = -\zeta \sigma_{\mathrm{AH}} E_x - \frac{\sigma_{\mathrm{F}}}{2e}\partial_z \mu_{\mathrm{s}}^{\mathrm{F}}, \qquad (9)$$

where σ_{F}, σ_{AH} are the longitudinal and anomalous Hall conductivities, ζ is the spin polarization of the anomalous Hall conductivity, $\theta_{\mathrm{AH}} = \sigma_{\mathrm{AH}}/\sigma_{\mathrm{F}}$ is the anomalous Hall angle of the NM layer, E_x is the electric field applied along the *x* direction, j_i^k is a charge current density flowing along *i* direction in *k* layer, $j_i^{s,k}$ is a spin current density flowing along *i* direction in *k* layer, and μ_s^{F} is the spin chemical potential polarized along the magnetization direction which satisfies the diffusion equation. Thus, we have

$$\mu_s^{\mathrm{F}} = A^{\mathrm{F}} e^{z/l_{\mathrm{sf}}^{\mathrm{F}}} + B^{\mathrm{F}} e^{-z/l_{\mathrm{sf}}^{\mathrm{F}}}, \qquad (10)$$

where A and B are constants determined by boundary conditions and $l_{\mathrm{sf}}^{\mathrm{F}}$ is the spin diffusion length of FM.

In the NM layer which includes the SHE, the charge and spin current densities are given by

$$j_x^{\mathrm{N}} = \sigma_{\mathrm{N}} E_x + \theta_{\mathrm{SH}} j_{z,y}^{\mathrm{s,N}}, \qquad j_{z,y}^{\mathrm{s,N}} = -\sigma_{\mathrm{SH}} E_x - \frac{\sigma_{\mathrm{N}}}{2e} \partial_z \mu_{s,y}^{\mathrm{N}}, \tag{11}$$

where σ_{N}, σ_{SH} are the longitudinal and spin Hall conductivities, $\theta_{\mathrm{SH}}(= \sigma_{\mathrm{SH}}/\sigma_{\mathrm{N}})$ is the spin Hall angle of the NM layer. From the diffusion equation of the spin chemical potential, $\mu_{s,i}^{\mathrm{N}}$, we have

$$\mu_{\mathrm{i}}^{\mathrm{N,s}} = A_{\mathrm{i}}^{\mathrm{N}} e^{z/l_{\mathrm{sf}}^{\mathrm{F}}} + B_{\mathrm{i}}^{\mathrm{N}} e^{-z/l_{\mathrm{sf}}^{\mathrm{F}}}. \tag{12}$$

At the NM/FM interface, the charge and spin currents are given by [6-8]

$$j_z(0^-) = -(G_\uparrow + G_\downarrow)\left(\frac{\Delta\mu}{e}\right) - (G_\uparrow - G_\downarrow)\left(\mathbf{m}\cdot\frac{\Delta\boldsymbol{\mu}^s}{e}\right), \tag{13}$$

$$\mathbf{j}_{z,\mathrm{T}}^{\mathrm{s}}(0^-) = \frac{1}{2e}[\mathrm{Re}(G_{\uparrow\downarrow})(2\Delta\boldsymbol{\mu}^{\mathrm{s}} \times \mathbf{m}) \times \mathbf{m} + \mathrm{Im}(G_{\uparrow\downarrow})(2\Delta\boldsymbol{\mu}^{\mathrm{s}} \times \mathbf{m})] + \mathbf{j}_{\mathrm{s,T}}^{\mathrm{ISOC}}(0^-), \tag{14}$$

$$j_{z,\mathrm{L}}^{\mathrm{s}}(0^-) = -\frac{1}{2e}[(G_\uparrow + G_\downarrow)(\mathbf{m}\cdot\Delta\boldsymbol{\mu}^{\mathrm{s}}) + (G_\uparrow - G_\downarrow)\Delta\mu] + j_{s,\mathrm{L}}^{\mathrm{ISOC}}(0^-), \tag{15}$$

where the subscripts T and L represent the transverse and longitudinal components, $G_\uparrow(G_\downarrow)$ is the interface conductivity of majority (minority) spin, $G_{\uparrow\downarrow}$ is the spin mixing conductance, and $\Delta\mu(\Delta\boldsymbol{\mu}^{\mathrm{s}})$ is the charge (spin) chemical potential drop over the interface, i.e. $\Delta\boldsymbol{\mu}^{\mathrm{s}} = \boldsymbol{\mu}^{\mathrm{F,s}}(0^+) - \boldsymbol{\mu}^{\mathrm{N,s}}(0^-)$. The new spin current from the interface spin-orbit interaction is introduced as $\mathbf{j}_{\mathrm{s}}^{\mathrm{ISOC}} = \boldsymbol{\sigma}^{\mathrm{ISOC}}(\mathbf{m})E_x$ [9,10].

The boundary conditions in the NM/FM structure are

$$j_z = 0, \qquad \mathbf{j}_z^{\mathrm{s}}(z = -t_{\mathrm{N}}) = 0, \qquad \mathbf{j}_z^{\mathrm{s}}(z = t_{\mathrm{F}}) = 0. \tag{16}$$

We note that these boundary conditions may need to be modified for thin NiFe films because a transverse spin current may survive at the outer surface of NiFe. This modification will change the magnitude of SMR [11]. However, we note that the sign of each SMR determined by each contribution is unchanged because the general symmetry argument in Supplementary Note 7 is valid regardless of the detailed boundary conditions. In addition, we observed a clear negative SMR signal for thicker t_{F} cases where the used boundary conditions are valid. Therefore, a possible modification of the boundary condition for thin t_{F} cases does not alter our main conclusion.

By solving Supplementary Equations (9-16) and averaging over the film thicknesses, we obtain the average charge current densities for $\mathbf{m} = \hat{\mathbf{z}}$,

$$j_x^{\mathrm{F}}(\mathbf{m} = \hat{\mathbf{z}}) = \sigma_{\mathrm{F}} E_x + j_{x,\mathrm{AH}}^{\mathrm{F}}(\mathbf{m} = \hat{\mathbf{z}}), \tag{17}$$

$$j_x^{\mathrm{N}}(\mathbf{m} = \hat{\mathbf{z}}) = \sigma_{\mathrm{N}} E_x + j_{x,\mathrm{SH}}^{\mathrm{N}}(\mathbf{m} = \hat{\mathbf{z}}) + j_{x,\mathrm{ISOC}}^{\mathrm{N}}(\mathbf{m} = \hat{\mathbf{z}}), \tag{18}$$

where

$$j_{x,\mathrm{AH}}^{\mathrm{F}}(\mathbf{m} = \hat{\mathbf{z}})/E_x = -\zeta^2 \sigma_{\mathrm{F}} \theta_{\mathrm{AH}}^2, \tag{19}$$

$$j_{x,\mathrm{SH}}^{\mathrm{N}}(\mathbf{m} = \hat{\mathbf{z}})/E_x = -\sigma_{\mathrm{N}} \theta_{\mathrm{SH}}^2 \left\{ \left[1 - \frac{2l_{\mathrm{sf}}^{\mathrm{N}}}{t_{\mathrm{N}}} \tanh\left(\frac{t_{\mathrm{N}}}{2l_{\mathrm{sf}}^{\mathrm{N}}}\right) \right] + \frac{l_{\mathrm{sf}}^{\mathrm{N}}}{t_{\mathrm{N}}} \mathrm{Re} \left[\frac{\tilde{G} \tanh^2\left(\frac{t_{\mathrm{N}}}{2l_{\mathrm{sf}}^{\mathrm{N}}}\right)}{1 + \tilde{G} \coth\left(\frac{t_{\mathrm{N}}}{l_{\mathrm{sf}}^{\mathrm{N}}}\right)} \right] \right\}, \tag{20}$$

$$j_{x,\mathrm{ISOC}}^{\mathrm{N}}(\mathbf{m} = \hat{\mathbf{z}})/E_x = -\theta_{\mathrm{SH}} \frac{l_{\mathrm{sf}}^{\mathrm{N}}}{t_{\mathrm{N}}} \left[1 - \mathrm{sech}\left(\frac{t_{\mathrm{N}}}{l_{\mathrm{sf}}^{\mathrm{N}}}\right) \right] \frac{\tilde{G}_{\mathrm{I}} \sigma_x^{\mathrm{ISOC}}(\mathbf{m}=\hat{\mathbf{z}}) - \left[\tilde{G}_{\mathrm{R}} + \tanh\left(\frac{t_{\mathrm{N}}}{l_{\mathrm{sf}}^{\mathrm{N}}}\right) \right] \sigma_y^{\mathrm{ISOC}}(\mathbf{m}=\hat{\mathbf{z}})}{\tilde{G}^2 + 2\tilde{G}_{\mathrm{R}} \tanh\left(\frac{t_{\mathrm{N}}}{l_{\mathrm{sf}}^{\mathrm{N}}}\right) + \tanh^2\left(\frac{t_{\mathrm{N}}}{l_{\mathrm{sf}}^{\mathrm{N}}}\right)}$$

$$\approx \theta_{\mathrm{SH}} \sigma_y^{\mathrm{ISOC}}(\mathbf{m} = \hat{\mathbf{z}}) \frac{l_{\mathrm{sf}}^{\mathrm{N}}}{t_{\mathrm{N}}} \frac{1 - \mathrm{sech}\left(\frac{t_{\mathrm{N}}}{l_{\mathrm{sf}}^{\mathrm{N}}}\right)}{\tilde{G} + \tanh\left(\frac{t_{\mathrm{N}}}{l_{\mathrm{sf}}^{\mathrm{N}}}\right)}, \tag{21}$$

where $j_{x,0}^{\mathrm{F}} = \sigma_{\mathrm{F}} E_x$ and $j_{x,0}^{\mathrm{N}} = \sigma_{\mathrm{N}} E_x$. Here we introduce the dimensionless quantities

$$\tilde{G} = G_{\uparrow\downarrow} \frac{2l_{\mathrm{sf}}^{\mathrm{N}}}{\sigma_{\mathrm{N}}}, \quad \tilde{G}_{\mathrm{R}} = \mathrm{Re}(G_{\uparrow\downarrow}) \frac{2l_{\mathrm{sf}}^{\mathrm{N}}}{\sigma_{\mathrm{N}}}, \quad \tilde{G}_{\mathrm{I}} = \mathrm{Im}(G_{\uparrow\downarrow}) \frac{2l_{\mathrm{sf}}^{\mathrm{N}}}{\sigma_{\mathrm{N}}}, \tag{22}$$

and we neglect $\mathrm{Im}(G_{\uparrow\downarrow})$ which is an order of magnitude smaller than $\mathrm{Re}(G_{\uparrow\downarrow})$.

For $\mathbf{m} = \hat{\mathbf{y}}$, we have

$$j_x^{\mathrm{F}}(\mathbf{m} = \hat{\mathbf{y}}) = \sigma_{\mathrm{F}} E_x + j_{x,\mathrm{AH}}^{\mathrm{F}}(\mathbf{m} = \hat{\mathbf{y}}) + j_{x,\mathrm{AS}}^{\mathrm{F}}(\mathbf{m} = \hat{\mathbf{y}}) + j_{x,\mathrm{ISOC}}^{\mathrm{F}}(\mathbf{m} = \hat{\mathbf{y}}), \tag{23}$$

$$j_x^{\mathrm{N}}(\mathbf{m} = \hat{\mathbf{y}}) = \sigma_{\mathrm{N}} E_x + j_{x,\mathrm{SH}}^{\mathrm{N}}(\mathbf{m} = \hat{\mathbf{y}}) + j_{x,\mathrm{AS}}^{\mathrm{N}}(\mathbf{m} = \hat{\mathbf{y}}) + j_{x,\mathrm{ISOC}}^{\mathrm{N}}(\mathbf{m} = \hat{\mathbf{y}}), \tag{24}$$

where the charge current components are

$$j_{x,\mathrm{AH}}^{\mathrm{F}}(\mathbf{m} = \hat{\mathbf{y}})/E_x = -\sigma_{\mathrm{F}} \zeta^2 \theta_{\mathrm{AH}}^2$$

$$+ \sigma_{\mathrm{F}} \zeta^2 \theta_{\mathrm{AH}}^2 \frac{4l_{\mathrm{sf}}^{\mathrm{F}}}{t_{\mathrm{F}}} \sinh\left(\frac{t_{\mathrm{N}}}{2l_{\mathrm{sf}}^{\mathrm{N}}}\right) \left\{ 2G_{\uparrow} G_{\downarrow} l_{\mathrm{sf}}^{\mathrm{F}} \rho_{\mathrm{F}} \cosh\left(\frac{t_{\mathrm{F}}}{2l_{\mathrm{sf}}^{\mathrm{F}}}\right) \sinh\left(\frac{t_{\mathrm{N}}}{l_{\mathrm{sf}}^{\mathrm{N}}}\right) \right.$$

$$\left. + \rho_{\mathrm{N}} \sinh\left(\frac{t_{\mathrm{F}}}{2l_{\mathrm{sf}}^{\mathrm{F}}}\right) \left[4G_{\uparrow} G_{\downarrow} l_{\mathrm{sf}}^{\mathrm{N}} \cosh\left(\frac{t_{\mathrm{N}}}{l_{\mathrm{sf}}^{\mathrm{N}}}\right) + (G_{\uparrow} + G_{\downarrow}) \sigma_{\mathrm{N}} \sinh\left(\frac{t_{\mathrm{N}}}{l_{\mathrm{sf}}^{\mathrm{N}}}\right) \right] \right\} / \mathcal{F}(t_{\mathrm{F}}), \tag{25}$$

$$j_{x,\mathrm{AS}}^{\mathrm{F}}(\mathbf{m} = \hat{\mathbf{y}})/E_x = -\zeta \theta_{\mathrm{AH}} \theta_{\mathrm{SH}} \frac{16l_{\mathrm{sf}}^{\mathrm{F}}}{t_{\mathrm{F}}} G_{\uparrow} G_{\downarrow} l_{\mathrm{sf}}^{\mathrm{N}} \sinh^2\left(\frac{t_{\mathrm{F}}}{2l_{\mathrm{sf}}^{\mathrm{F}}}\right) \sinh^2\left(\frac{t_{\mathrm{N}}}{2l_{\mathrm{sf}}^{\mathrm{N}}}\right) / \mathcal{F}(t_{\mathrm{F}}), \tag{26}$$

$$j_{x,\mathrm{ISOC}}^{\mathrm{F}}(\mathbf{m} = \hat{\mathbf{y}})/E_x = \zeta \theta_{\mathrm{AH}} \sigma_y^{\mathrm{ISOC}}(\mathbf{m} = \hat{\mathbf{y}}) \frac{2l_{\mathrm{sf}}^{\mathrm{F}}}{t_{\mathrm{F}}} (G_{\uparrow} + G_{\downarrow}) \sinh^2\left(\frac{t_{\mathrm{F}}}{2l_{\mathrm{sf}}^{\mathrm{F}}}\right) \sinh\left(\frac{t_{\mathrm{N}}}{l_{\mathrm{sf}}^{\mathrm{N}}}\right) / \mathcal{F}(t_{\mathrm{F}}), \tag{27}$$

$$j_{x,\mathrm{SH}}^{\mathrm{N}}(\mathbf{m} = \hat{\mathbf{y}})/E_x = -\sigma_{\mathrm{N}} \theta_{\mathrm{SH}}^2 + \sigma_{\mathrm{N}} \theta_{\mathrm{SH}}^2 \frac{4l_{\mathrm{sf}}^{\mathrm{N}}}{t_{\mathrm{N}}} \sinh\left(\frac{t_{\mathrm{N}}}{2l_{\mathrm{sf}}^{\mathrm{N}}}\right) \left[4G_{\uparrow} G_{\downarrow} \rho_{\mathrm{F}} l_{\mathrm{sf}}^{\mathrm{F}} \cosh\left(\frac{t_{\mathrm{F}}}{l_{\mathrm{sf}}^{\mathrm{F}}}\right) \sinh\left(\frac{t_{\mathrm{N}}}{2l_{\mathrm{sf}}^{\mathrm{N}}}\right) \right.$$

$$+\rho_{\mathrm{N}}\sinh\left(\frac{t_{\mathrm{F}}}{l_{\mathrm{sf}}^{\mathrm{F}}}\right)\left(2G_{\uparrow}G_{\downarrow}l_{\mathrm{sf}}^{\mathrm{N}}\cosh\left(\frac{t_{\mathrm{N}}}{2l_{\mathrm{sf}}^{\mathrm{N}}}\right)+(G_{\uparrow}+G_{\downarrow})\,\sigma_{\mathrm{N}}\sinh\left(\frac{t_{\mathrm{N}}}{2l_{\mathrm{sf}}^{\mathrm{N}}}\right)\right)\Big]/\mathcal{F}(t_{\mathrm{F}}),\quad(28)$$

$$j_{x,\mathrm{AS}}^{\mathrm{N}}(\mathbf{m}=\hat{\mathbf{y}})/E_x=-\zeta\theta_{\mathrm{AH}}\theta_{\mathrm{SH}}\frac{16l_{\mathrm{sf}}^{\mathrm{N}}}{t_{\mathrm{N}}}G_{\uparrow}G_{\downarrow}l_{\mathrm{sf}}^{\mathrm{F}}\sinh^2\left(\frac{t_{\mathrm{F}}}{2l_{\mathrm{sf}}^{\mathrm{F}}}\right)\sinh^2\left(\frac{t_{\mathrm{N}}}{2l_{\mathrm{sf}}^{\mathrm{N}}}\right)/\mathcal{F}(t_{\mathrm{F}}),\quad(29)$$

$$j_{x,\mathrm{ISOC}}^{\mathrm{N}}(\mathbf{m}=\hat{\mathbf{y}})/E_x=\theta_{\mathrm{SH}}\sigma_y^{\mathrm{ISOC}}(\mathbf{m}=\hat{\mathbf{y}})\frac{2l_{\mathrm{sf}}^{\mathrm{N}}}{t_{\mathrm{N}}}(G_{\uparrow}+G_{\downarrow})\sinh\left(\frac{t_{\mathrm{F}}}{l_{\mathrm{sf}}^{\mathrm{F}}}\right)\sinh^2\left(\frac{t_{\mathrm{N}}}{2l_{\mathrm{sf}}^{\mathrm{N}}}\right)/\mathcal{F}(t_{\mathrm{F}}),\quad(30)$$

and

$$\mathcal{F}(t_{\mathrm{F}})=4G_{\uparrow}G_{\downarrow}\rho_{\mathrm{F}}l_{\mathrm{sf}}^{\mathrm{F}}\cosh\left(\frac{t_{\mathrm{F}}}{l_{\mathrm{sf}}^{\mathrm{F}}}\right)\sinh\left(\frac{t_{\mathrm{N}}}{2l_{\mathrm{sf}}^{\mathrm{N}}}\right)$$

$$+\rho_{\mathrm{N}}\sinh\left(\frac{t_{\mathrm{F}}}{l_{\mathrm{sf}}^{\mathrm{F}}}\right)\left[2G_{\uparrow}G_{\downarrow}l_{\mathrm{sf}}^{\mathrm{N}}\cosh\left(\frac{t_{\mathrm{N}}}{l_{\mathrm{sf}}^{\mathrm{N}}}\right)+(G_{\uparrow}+G_{\downarrow})\,\sigma_{\mathrm{N}}\sinh\left(\frac{t_{\mathrm{N}}}{l_{\mathrm{sf}}^{\mathrm{N}}}\right)\right].\quad(31)$$

The subscript *SH* represents that the charge current is generated by the interconversion processes from SHE and inverse SHE of NM. The resulting charge current is proportional to θ_{SH}^2. *AH* represents that the charge current is generated by the interconversions from AHE and its inverse effect in FM layer. The resulting charge current is proportional to θ_{AH}^2. *AS* represents the charge current originating from the combined effect of AHE and SHE, which is proportional to $\theta_{\mathrm{AH}}\theta_{\mathrm{SH}}$. *ISOC* indicates the charge current originating from the ISOC effect.

The new spin currents in Supplementary Equations (14) and (15) are generated by the charge-to-spin conversion process from the interfacial spin-orbit scattering. From the Onsager reciprocity relation, we read the spin-to-charge conversion from the interfacial spin-orbit scattering:

$$j_x^{\mathrm{ISOC}}=\frac{\boldsymbol{\sigma}^{\mathrm{ISOC}}(-\hat{\mathbf{m}})}{L_z}\cdot\left(-\frac{\Delta\boldsymbol{\mu}^{\mathrm{s}}}{2e}\right),\quad(32)$$

where L_z is the length scale of interface generated charge current. We note that the L_z dependence of the charge current disappears after averaging over film thickness. Therefore, the resulting magnetoresistance does not depend on the choice of L_z. By solving Supplementary Equations (9-16) and (32), we have

$$j_x^{\mathrm{ISOC}}(\mathbf{m}=\hat{\mathbf{z}})=j_{x,\mathrm{ISOC}}^{\mathrm{SH}}(\mathbf{m}=\hat{\mathbf{z}})+j_{x,\mathrm{ISOC}}^{\mathrm{ISOC}}(\mathbf{m}=\hat{\mathbf{z}}),\quad(33)$$

$$j_x^{\mathrm{ISOC}}(\mathbf{m}=\hat{\mathbf{y}})=j_{x,\mathrm{ISOC}}^{\mathrm{SH}}(\mathbf{m}=\hat{\mathbf{y}})+j_{x,\mathrm{ISOC}}^{\mathrm{AH}}(\mathbf{m}=\hat{\mathbf{y}})+j_{x,\mathrm{ISOC}}^{\mathrm{ISOC}}(\mathbf{m}=\hat{\mathbf{y}}),\quad(34)$$

$$j_{x,\mathrm{ISOC}}^{\mathrm{AH}}(\mathbf{m}=\hat{\mathbf{y}})/E_x=-\zeta\theta_{\mathrm{AH}}\sigma_y^{\mathrm{ISOC}}(\mathbf{m}=\hat{\mathbf{y}})\frac{2l_{\mathrm{sf}}^{\mathrm{F}}}{t_{\mathrm{F}}}(G_{\uparrow}+G_{\downarrow})\sinh^2\left(\frac{t_{\mathrm{F}}}{2l_{\mathrm{sf}}^{\mathrm{F}}}\right)\sinh\left(\frac{t_{\mathrm{N}}}{l_{\mathrm{sf}}^{\mathrm{N}}}\right)/\mathcal{F}(t_{\mathrm{F}})$$

$$=-j_{x,\mathrm{ISOC}}^{\mathrm{F}}(\mathbf{m}=\hat{\mathbf{y}})/E_x\quad(35)$$

$$j_{x,\mathrm{ISOC}}^{\mathrm{SH}}(\mathbf{m}=\hat{\mathbf{z}})/E_x\approx-\theta_{\mathrm{SH}}\sigma_y^{\mathrm{ISOC}}(\mathbf{m}=\hat{\mathbf{z}})\frac{l_{\mathrm{sf}}^{\mathrm{N}}}{t_{\mathrm{N}}}\frac{1-\mathrm{sech}\left(\frac{t_{\mathrm{N}}}{l_{\mathrm{sf}}^{\mathrm{N}}}\right)}{\tilde{G}+\tanh\left(\frac{t_{\mathrm{N}}}{l_{\mathrm{sf}}^{\mathrm{N}}}\right)}$$

$$= -j_{x,\text{ISOC}}^{\text{N}}(\mathbf{m} = \hat{\mathbf{z}})/E_x \tag{36}$$

$$j_{x,\text{ISOC}}^{\text{SH}}(\mathbf{m} = \hat{\mathbf{y}})/E_x = -\theta_{\text{SH}}\sigma_y^{\text{ISOC}}(\mathbf{m} = \hat{\mathbf{y}})\frac{2l_{\text{sf}}^{\text{N}}}{t_{\text{N}}}(G_\uparrow + G_\downarrow)\sinh\left(\frac{t_{\text{F}}}{l_{\text{sf}}^{\text{F}}}\right)\sinh^2\left(\frac{t_{\text{N}}}{2l_{\text{sf}}^{\text{N}}}\right)/\mathcal{F}(t_{\text{F}}),$$

$$= -j_{x,\text{ISOC}}^{\text{N}}(\mathbf{m} = \hat{\mathbf{y}})/E_x \tag{37}$$

$$j_{x,\text{ISOC}}^{\text{ISOC}}(\mathbf{m} = \hat{\mathbf{z}})/E_x \approx -\frac{l_{\text{sf}}^{\text{N}}}{\sigma_{\text{N}}t_{\text{N}}}\frac{\left[\sigma_y^{\text{ISOC}}(\mathbf{m}=\hat{\mathbf{z}})\right]^2-\left[\sigma_x^{\text{ISOC}}(\mathbf{m}=\hat{\mathbf{z}})\right]^2}{\tilde{G}+\tanh\left(\frac{t_{\text{N}}}{l_{\text{sf}}^{\text{N}}}\right)}, \tag{38}$$

$$j_{x,\text{ISOC}}^{\text{ISOC}}(\mathbf{m} = \hat{\mathbf{y}})/E_x = -\left[\sigma_y^{\text{ISOC}}(\mathbf{m} = \hat{\mathbf{y}})\right]^2\frac{(G_\uparrow+G_\downarrow)}{t_{\text{N}}}$$

$$\times\left[\rho_{\text{N}}l_{\text{sf}}^{\text{N}}\cosh\left(\frac{t_{\text{N}}}{l_{\text{sf}}^{\text{N}}}\right)\sinh\left(\frac{t_{\text{F}}}{l_{\text{sf}}^{\text{F}}}\right) + \rho_{\text{F}}l_{\text{sf}}^{\text{F}}\cosh\left(\frac{t_{\text{F}}}{l_{\text{sf}}^{\text{F}}}\right)\sinh\left(\frac{t_{\text{N}}}{l_{\text{sf}}^{\text{N}}}\right)\right]/\mathcal{F}(t_{\text{F}}). \tag{39}$$

We note that we choose L_z as t_{F} for Supplementary Equation (35), and we choose L_z as t_{N} for Supplementary Equations (36-39). Supplementary Equation (35) describes the hybridization of interfacial spin-orbit scattering and AHE (charge-to-spin conversion by AHE and spin-to-charge conversion by ISOC). As discussed in Supplementary Note 7, this contribution is exactly cancelled by its reciprocal effect in Supplementary Equation (27) (charge-to-spin conversion by ISOC and spin-to-charge conversions by AHE). Supplementary Equations (36) and (37) describe the hybridization of SHE and interfacial spin-orbit scattering. These effects are also cancelled by their reciprocal effect described in Supplementary Equations (21) and (30). The charge currents in Supplementary Equations (38) and (39) originate from the spin-to-charge and charge-to-spin conversions by ISOC. The remaining components which contribute to the magnetoresistance of the FM/NM bilayer are $j_{x,\text{AH}}^{\text{F}}$, $j_{x,\text{SH}}^{\text{N}}$, $j_{x,\text{AS}}^{\text{F,N}}$, and $j_{x,\text{ISOC}}^{\text{ISOC}}$.

B. Calculation of the magnetoresistance

In the magnetoresistance experiments, the total resistance of FM and NM layers is the observable quantity. There are various magnetoresistance contributions from different physical processes including GSE in ferromagnetic layer. However, the resistance change from the GSE is not of our interest. By using the method in Supplementary Reference [12], we remove the resistance change of the single ferromagnetic layer including GSE contribution. This calibration method is based on the parallel circuit model and somewhat crude. Therefore, the quantitative description of the magnetoresistance is difficult in our model calculation. However, this does not alter our main conclusion (existence of the additional source in the negative Δr_{MR}) as discuss in Supplementary Note 7. Then, we define the calibrated MR as

$$\Delta r_{\rm MR}^{\rm cal} = \frac{\Delta R^{\rm cal}}{R_0} = \Delta r_{\rm raw} - \frac{R_0}{R_{\rm F}}\frac{\Delta R_{\rm F,SL}}{R_{\rm F}}$$

$$= \frac{R_0}{R_{\rm N}}\left[\frac{\Delta R_{\rm N}}{R_{\rm N}}\right] + \frac{R_0}{R_{\rm F}}\left[\frac{\Delta R_{\rm F} - \Delta R_{\rm F}^{\rm AHE} - \Delta R_{\rm F}^{\rm GSE}}{R_{\rm F}}\right]$$

$$= \frac{R_0}{R_{\rm N}}\left[\frac{\Delta R_{\rm N}^{\rm SH} + \Delta R_{\rm N}^{\rm AS} + \Delta R_{\rm I}^{\rm ISOC}}{R_{\rm N}}\right] + \frac{R_0}{R_{\rm F}}\left[\frac{\Delta R_{\rm F}^{\rm AS} + \Delta R_{\rm I}^{\rm ISOC}}{R_{\rm F}}\right], \quad (40)$$

In a compact form, we write

$$\Delta r_{\rm MR}^{\rm cal} = \Delta r_{\rm N}^{\rm SH} + \Delta r_{\rm N}^{\rm AS} + \Delta r_{\rm N}^{\rm ISOC} + \Delta r_{\rm F}^{\rm AS} + \Delta r_{\rm F}^{\rm ISOC} \quad (41)$$

where

$$\Delta r_{\rm N}^{\rm SH} = \frac{R_0}{R_{\rm N}}\frac{\Delta R_{\rm N}^{\rm SH}}{R_{\rm N}}, \qquad \Delta r_{\rm N}^{\rm AS} = \frac{R_0}{R_{\rm N}}\frac{\Delta R_{\rm N}^{\rm AS}}{R_{\rm N}}, \qquad \Delta r_{\rm N}^{\rm ISOC} = \frac{R_0}{R_{\rm N}}\frac{\Delta R_{\rm I}^{\rm ISOC}}{R_{\rm N}}$$

$$\Delta r_{\rm F}^{\rm AS} = \frac{R_0}{R_{\rm F}}\frac{\Delta R_{\rm F}^{\rm AS}}{R_{\rm F}}, \qquad \Delta r_{\rm F}^{\rm ISOC} = \frac{R_0}{R_{\rm F}}\frac{\Delta R_{\rm I}^{\rm ISOC}}{R_{\rm F}}.$$

$\Delta r_{\rm raw}$ is the raw result of the magnetoresistances ratio and the subscript F(N) stands for ferromagnet (heavy metal) layer. $\Delta R_{\rm F}^{\rm AHE}$ is the resistance change from the interconversion process generated by the AHE which is proportional to $\theta_{\rm AH}^2$. $\Delta R_{\rm F}^{\rm GSE}$ is the resistance change from the GSE. The full expressions for the remaining resistance components are obtained by inserting above equations to the following Eq.:

$$\frac{\Delta R_{\rm N}^{\rm SH}}{R_{\rm N}} = \frac{j_{x,\rm SH}^{\rm N}(\mathbf{m}=\hat{\mathbf{y}}) - j_{x,\rm SH}^{\rm N}(\mathbf{m}=\hat{\mathbf{z}})}{j_{x,0}^{\rm N}},$$

$$\frac{\Delta R_{\rm N}^{\rm AS}}{R_{\rm N}} = \frac{j_{x,\rm AS}^{\rm N}(\mathbf{m}=\hat{\mathbf{y}})}{j_{x,0}^{\rm N}}, \quad \frac{\Delta R_{\rm F}^{\rm AS}}{R_{\rm F}} = \frac{j_{x,\rm AS}^{\rm F}(\mathbf{m}=\hat{\mathbf{y}})}{j_{x,0}^{\rm F}},$$

$$\frac{\Delta R_{\rm I}^{\rm ISOC}}{R_{\rm N,F}} = \frac{j_x^{\rm ISOC}(\mathbf{m}=\hat{\mathbf{y}}) - j_x^{\rm ISOC}(\mathbf{m}=\hat{\mathbf{z}})}{j_{x,0}^{\rm N,F}}, \quad (42)$$

where $\Delta R_{\rm N}^{\rm SH}$ is the magnetoresistance contribution from the spin Hall effect in NM layer. In the transparent interface limit, $\Delta R_{\rm N}^{\rm SH}$ is equivalent to the SMR expression in the metallic bilayer structure of NM/FM [13]. In this metallic limit, the ISOC effects on the magnetoresistance vanish because the interface is transparent. If we neglect the longitudinal

spin current transmission between NM and FM layers, $\Delta R_{\mathrm{N}}^{\mathrm{SH}}$ corresponds to SMR expression in NM/ferromagnetic insulator (FI) structure [14]. In both limits, $\Delta R_{\mathrm{N}}^{\mathrm{SH}}$ gives positive contribution to the Δr_{MR}. $\Delta R_{\mathrm{N}}^{\mathrm{AS}}$ and $\Delta R_{\mathrm{F}}^{\mathrm{AS}}$ are generated by the combined effect of AHE and SHE. The signs of $\Delta R_{\mathrm{N}}^{\mathrm{AS}}$ and $\Delta R_{\mathrm{F}}^{\mathrm{AS}}$ are determined by $\zeta\theta_{\mathrm{AH}}\theta_{\mathrm{SH}}$. In our experiment, θ_{AH} is negative in NiFe ($\theta_{\mathrm{AH}} = -0.0033$). Therefore, $\Delta R_{\mathrm{N}}^{\mathrm{AS}}$ and $\Delta R_{\mathrm{F}}^{\mathrm{AS}}$ give positive contributions to the NiFe/Ta sample ($\theta_{\mathrm{SH}} < 0$) and negative contribution to the NiFe/Pt sample ($\theta_{\mathrm{SH}} > 0$). The effects of these terms are however negligible because of the small anomalous Hall angle in our experiment (see Supplementary Figure 7).

Therefore, the negative values of calibrated MR in Ta/NiFe samples cannot be explained without interfacial spin-orbit scattering effect in $\Delta R_{\mathrm{I}}^{\mathrm{ISOC}}$. In Supplementary Figure 7, we show the full calculation results of the calibrated MR. In the calculation, we use $l_{\mathrm{sf}}^{\mathrm{Ta}} = 1$ nm, $G_{\uparrow\downarrow,\mathrm{NiFe/Ta}} = 14.4 \times 10^{14}$ $\Omega^{-1}\mathrm{m}^{-2}$ [15], $\rho_{\mathrm{Ta}} = 300$ μΩcm, $\theta_{\mathrm{SH,Ta}} = -0.1$, $\zeta = 5$ [3], $\theta_{\mathrm{AH}} = -0.0033$, $G_{\uparrow} = 5 \times 10^{13}$ $\Omega^{-1}\mathrm{m}^{-2}$, and $G_{\downarrow} = 3.5 \times 10^{13}$ $\Omega^{-1}\mathrm{m}^{-2}$. We do not fit the positive Δr_{MR} of the Pt/NiFe samples, because there are several positive contributions, which are uneasy to disentangle. For the fitting, we assume $\sigma_{\mathrm{ISOC}}^{y}$ varies with FM thickness t_{F} (the inset of Supplementary Figure7a), motivated by theoretical prediction [9,10] that the ISOC effect depends on the relative scattering strengths in FM and NM, which would vary with t_{F} because of the surface scattering.

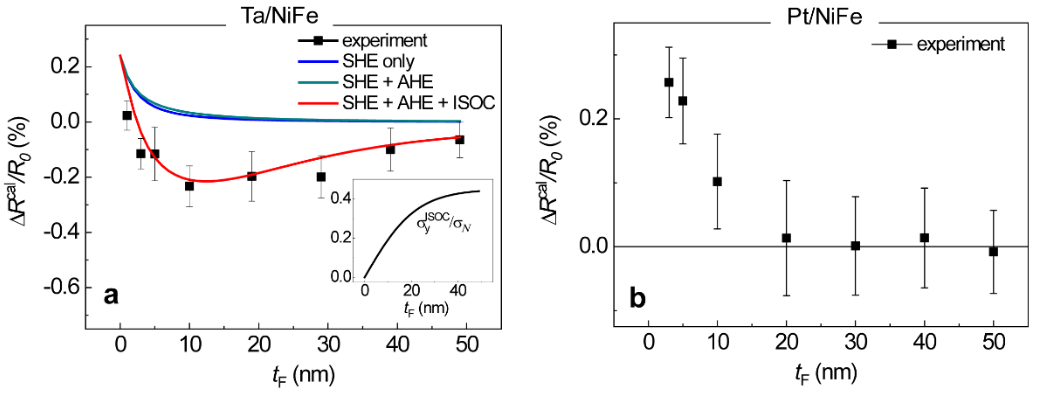

Supplementary Figure 7. Thickness dependence of the calibrated Δr_{MR}. a,b, Calibrated $\Delta r_{\mathrm{MR}}^{\mathrm{cal}}$ in Ta/NiFe sample (**a**) and Pt/NiFe sample (**b**). The experimental values are denoted in squares. Solid lines are fitting results including only SHE (blue), SHE and AHE (green), and SHE, AHE and ISOC effect (red). Inset in **a** shows the thickness dependence of the ISOC effect,

which we use for the fitting. The error bars in a – b are from the fitting to determine.

Because the full expressions of the resistance components in the NM/FM bilayer system is too complicated, we consider a limit which neglects the longitudinal spin current transmission between NM and FM layers. In this limit, the resistance changes from the combined effect of AHE and SHE ($\Delta R_{\mathrm{N}}^{\mathrm{AS}}$, $\Delta R_{\mathrm{F}}^{\mathrm{AS}}$) vanish. The remaining components of the ΔR_{MR} contributions are

$$\Delta r_{\mathrm{N}}^{\mathrm{SH}} = \theta_{\mathrm{SH}}^{2} \frac{\rho_{\mathrm{F}} l_{\mathrm{sf}}^{\mathrm{N}}}{\rho_{\mathrm{F}} t_{\mathrm{N}} + \rho_{\mathrm{N}} t_{\mathrm{F}}} \frac{\tilde{G} \tanh^{2}\left(\frac{t_{\mathrm{N}}}{2 l_{\mathrm{sf}}^{\mathrm{N}}}\right)}{1 + \tilde{G} \coth\left(\frac{t_{\mathrm{N}}}{l_{\mathrm{sf}}^{\mathrm{N}}}\right)}, \tag{43}$$

$$\Delta r_{\mathrm{N}}^{\mathrm{ISOC}} = -\frac{\rho_{\mathrm{F}}}{\rho_{\mathrm{F}} t_{\mathrm{N}} + \rho_{\mathrm{N}} t_{\mathrm{F}}} \left\{ \left[\sigma_{\mathrm{ISOC}}^{y}(\mathbf{m} = \hat{\mathbf{y}})\right]^{2} \left[\rho_{\mathrm{N}} l_{\mathrm{sf}}^{\mathrm{N}} \coth\left(\frac{t_{\mathrm{N}}}{l_{\mathrm{sf}}^{\mathrm{N}}}\right) + \rho_{\mathrm{F}} l_{\mathrm{sf}}^{\mathrm{F}} \coth\left(\frac{t_{\mathrm{F}}}{l_{\mathrm{sf}}^{\mathrm{F}}}\right)\right] - \rho_{\mathrm{N}} l_{\mathrm{sf}}^{\mathrm{N}} \frac{\left[\sigma_{\mathrm{ISOC}}^{y}(\mathbf{m} = \hat{\mathbf{z}})\right]^{2} - \left[\sigma_{\mathrm{ISOC}}^{x}(\mathbf{m} = \hat{\mathbf{z}})\right]^{2}}{\tilde{G} + \tanh\left(\frac{t_{\mathrm{N}}}{l_{\mathrm{sf}}^{\mathrm{N}}}\right)} \right\}. \tag{44}$$

From Supplementary Equation (43), we read that the positive SMR originating from the bulk spin Hall effect of NM decreases as the resistivity of NM increases. It is because less current flows through the NM layer as the resistivity of NM increases. The first term in Supplementary Equation (44) is always negative and the second term is conditionally negative when $\sigma_{\mathrm{ISOC}}^{x}(\mathbf{m} = \hat{\mathbf{z}}) \geq \sigma_{\mathrm{ISOC}}^{y}(\mathbf{m} = \hat{\mathbf{z}})$. Because $\tilde{G}(= 2 G_{\uparrow\downarrow} l_{\mathrm{sf}}^{\mathrm{N}} \rho_{\mathrm{N}})$ is proportional to the resistivity of NM layer, the first term of Supplementary Equation (44) would be dominant over the second term of Supplementary Equation (44) for the high resistive NM layer (such as Ta). The negative SMR monotonically increases with the spin diffusion length of NM ($l_{\mathrm{sf}}^{\mathrm{N}}$) and FM ($l_{\mathrm{sf}}^{\mathrm{F}}$). Because of the $\coth\left(\frac{t_{\mathrm{N}}}{l_{\mathrm{sf}}^{\mathrm{N}}}\right)$ dependence, it seems that $\Delta r_{\mathrm{N}}^{\mathrm{ISOC}}$ diverges for $t_{\mathrm{N}} \to 0$ and exponentially decreases as t_{N} increases. However, for $t_{\mathrm{N}} \to 0$, there is no FM/NM interface and $\sigma_{\mathrm{ISOC}}^{y}$ also vanishes. Thus, the resulting SMR is zero at $t_{\mathrm{N}} = 0$, has a maximum value for a finite t_{N}, and it decreases as t_{N} increases (the same argument also holds for t_{F}).

We suggest following approaches to observe the negative SMR in NM/FM bilayers. As

clearly seen in Supplementary Equation (44), the negative SMR increases in magnitude with increasing $\sigma_{\mathrm{ISOC}}^{y}$ (i.e., spin-orbit filtering contribution) and/or $\sigma_{\mathrm{ISOC}}^{x}$ (i.e., spin-orbit precession contribution). As σ_{ISOC} increases with the Rashba constant, which is known to be strongly related to the work function difference across the interface [16], a possible way to increase the negative SMR would be to use NM/FM bilayers with a large difference in the work function between NM and FM. Another way is to maximize the ISOC contribution is to increase the spin diffusion length of NM ($l_{\mathrm{sf}}^{\mathrm{N}}$) and FM ($l_{\mathrm{sf}}^{\mathrm{F}}$), because the above equation shows that the negative SMR in bilayers with fixed t_{N} and t_{F} monotonically increases in magnitude with $l_{\mathrm{sf}}^{\mathrm{N}}$ and $l_{\mathrm{sf}}^{\mathrm{F}}$. An alternative way to increase the negative SMR is to use a NM layer with a high resistivity. It is because a less current flows through the NM layer as the resistivity of NM increases. It results in the reduction of the positive SMR originating from the bulk spin Hall effect of NM.

Supplementary References

1. Demasius, K.-U., Phung, T., Zhang W., Hughes, B. P., Yang, S.-H., Kellock, A., Han, W., Pushp, A. & Parkin, S. S. P. Enhanced spin–orbit torques by oxygen incorporation in tungsten films, *Nat. Commun.* **7**,10644 (2016).

2. Avci, C. O., Garello, K., Gabureac, M., Ghosh, A., Fuhrer, A., Alvarado, S. F., & Gambardella, P. Interplay of spin-orbit torque and thermoelectric effects in ferromagnet/normal-metal bilayers. *Phys. Rev. B*, **90**, 224427 (2014).

3. Taniguchi, T., Grollier, J. & Stiles, M. D. Spin-transfer torques generated by the anomalous Hall effect and anisotropic magnetoresistance. *Phys. Rev. Applied* **3**, 044001 (2015).

4. Yang, Y. *et al.* Anomalous Hall magnetoresistance in a ferromagnet. *Nat. Commun.* **9**, 2255, (2018).

5. Amin, V. P., Zemen, J. & Stiles, M. D. Interface-generated spin currents. *Phys. Rev. Lett.* **121**, 136805 (2018).

6. Brataas, A., Nazarov, Y. V. & Bauer, G. E. Finite-element theory of transport in ferromagnet–normal metal systems. *Phys. Rev. Lett.* **84**, 2481 (2000).

7. Tserkovnyak, Y., Brataas, A. & Bauer, G. E. Spin pumping and magnetization dynamics in metallic multilayers. *Phys. Rev. B* **66**, 224403 (2002).

8. Polianski, M. & Brouwer, P. Current-induced transverse spin-wave instability in a thin nanomagnet. *Phys. Rev. Lett.* **92**, 026602 (2004).

9. Amin, V. & Stiles, M. Spin transport at interfaces with spin-orbit coupling: Formalism. *Phys. Rev. B* **94**, 104419 (2016).

10. Amin, V. P. & Stiles, M. D. Spin transport at interfaces with spin-orbit coupling: Phenomenology. *Phys. Rev. B* **94**, 104420 (2016).

11. Choi, J.-G., Lee, J. W. & Park, B.-G. Spin Hall magnetoresistance in heavy-metal/metallic-ferromagnet multilayer structures. *Phys. Rev. B* **96**, 174412 (2017).

12. Du, Y. *et al.* Anomalous spin orbit torques with large Rashba spin orbit coupling in epitaxial Pt/Co bilayers. *arXiv:1807.10867* (2018).

13. Kim, J., Sheng, P., Takahashi, S., Mitani, S. & Hayashi, M. Spin Hall magnetoresistance in metallic bilayers. *Phys. Rev. Lett.* **116**, 097201 (2016).

14. Chen, Y.-T. *et al.* Theory of spin Hall magnetoresistance. *Phys. Rev. B* **87**, 144411 (2013).

15. Weiler, M., Shaw, J. M., Nembach, H. T. & Silva, T. J. Detection of the DC inverse spin Hall effect due to spin pumping in a novel meander-stripline geometry. *IEEE Magn. Lett.* **5**, 1-4 (2014).

16. Tsai, H., Karube, S., Kondou, K., Yamaguchi, N., Ishii, F. & Otani, Y. Clear variation of spin splitting by changing electron distribution at non-magnetic metal/Bi_2O_3 interfaces. *Sci. Rep.* **8**, 1-8 (2018).
